# Mapping the research landscape of myelodysplastic syndromes: a scientometric analysis (2014–2025)

**DOI:** 10.1007/s10238-026-02226-z

**Published:** 2026-06-25

**Authors:** Jieyun Li, Wei Song Seetoh, Hao Xu, Yuchen Tao, Milin Liu, Shuyang Cai, Jiahui Lu, Cuncun Lu

**Affiliations:** 1 Institute of Hematology, Shanghai Municipal Hospital of Traditional Chinese Medicine, Shanghai University of Traditional Chinese Medicine, Shanghai, China; 2 Clinical Hematology Center, Shanghai Municipal Hospital of Traditional Chinese Medicine, Shanghai University of Traditional Chinese Medicine, Shanghai, China; 3 School of Biological Sciences, Nanyang Technological University, Singapore, Singapore; 4 Institute of Traditional Chinese and Western Medicine, Gansu University of Chinese Medicine, Lanzhou, Gansu China

**Keywords:** Myelodysplastic syndrome(s), Myelodysplastic neoplasm(s), Scientometric analysis, Precision medicine, Collaboration networks

## Abstract

This scientometric study critically evaluates the global evolution of myelodysplastic syndromes (MDS) research from 2014 to 2025, aiming to integrate fragmented knowledge and identify emerging trends. A scientometric analysis was conducted using data retrieved from the Web of Science Core Collection on August 2, 2025. The search was restricted to “article” and “review” in English containing “Myelodysplastic syndrome” or “Myelodysplastic syndromes” in the abstract, covering the period from January 1, 2014 onward. Scientometric tools (VOSviewer, CiteSpace, R-bibliometrix) were employed to analyze annual publication trends, country/institution/author contributions, collaboration networks, journal distribution, reference co-citation clusters, emerging research topics. Analysis of 6,822 publications revealed that global MDS research output peaked in 2021. The USA dominated with 2,838 publications, followed by China (1,169) and Germany (717), while top institutions (e.g., University of Texas MD Anderson Cancer Center, Memorial Sloan Kettering Cancer Center) and prolific authors (e.g., Garcia-Manero G, Kantarjian HM) were predominantly USA-based. Collaboration networks revealed a core-periphery structure with strong transatlantic ties and emerging Asian contributions. Thematic analysis identified research hotspots across multiple dimensions, including *5q deletion*, *trisomy 8*, *germline predisposition*, *TP53 mutation*, *clonal hematopoiesis*, *inflammation*, *venetoclax*, *luspatercept*, *targeted therapy*, *immunotherapy*, *pediatric population*, *VEXAS syndrome*, *IPSS-R*, *relapse*, *prognosis*, *overall survival*, and *artificial intelligence*. MDS research is shifting toward precision medicine. Future advances are expected to rely on multi-ethnic cohort building, mechanism exploration, combined treatment regimen optimization and novel drug development. Strengthening multidisciplinary collaboration is anticipated to facilitate clinical translation and foster more precise, equitable and clinically practical management of MDS globally.

**Supplementary Information:**

The online version contains supplementary material available at https://doi.org/10.1007/s10238-026-02226-z.

## Introduction

Myelodysplastic syndromes (MDS) represent a heterogeneous group of clonal hematopoietic stem cell disorders, primarily characterized by ineffective hematopoiesis, peripheral blood cytopenias, and a variable risk of progression to acute myeloid leukemia (AML) [1, 2]. MDS exhibits substantial variability in clinical manifestations and prognosis, spanning from indolent low-risk patients reliant on supportive care to aggressive high-risk cases with limited therapeutic options, underscoring the inherent complexity in diagnosis and management [1].

Recent advances including the development of diagnostic frameworks (World Health Organization[WHO] [3] and International Consensus Classification [ICC] [4), risk stratification models (Revised International Prognostic Scoring System [IPSS-R] [5] and Molecular International Prognostic Scoring System [IPSS-M] [6], and targeted therapeutics (hypomethylating agents [HMAs] [7] and lenalidomide [8, 9], have significantly advanced the field. However, critical challenges persist: incomplete molecular stratification failing to resolve prognostic ambiguity and clonal heterogeneity [6, 10], heterogeneous, non-durable standard-of-care responses with frequent primary or secondary resistance [11, 12], and the lack of robust, clinically validated integrated tools to translate multi-omics into actionable personalized strategies [13, 14]. These gaps continue to limit the precision of risk assessment and long-term therapeutic benefits for MDS patients [1, 14].

Over the past decade, MDS research has expanded rapidly, with intensive efforts focused on mechanistic exploration and clinical translation [1, 15]. Yet the global research landscape remains diverse [12, 16], largely attributed to the disease’s profound biological heterogeneity and incompletely elucidated pathogenic mechanisms [1]. To address these unresolved challenges, global research groups adopt divergent, independent investigative strategies to explore pathogenesis and develop optimal diagnostic and therapeutic modalities balancing efficacy, safety, and economic feasibility [17, 18]. Such scattered efforts impede the synthesis of global evidence and hinder cross-disciplinary translational collaboration.

Scientometrics provides a quantitative and visualized framework to trace disciplinary evolution [19]. By mapping co-authorship, co-citation and co-occurrence networks, it systematically identifies core knowledge domains, collaborative patterns and emerging research frontiers, offering a more objective portrayal of the research landscape than conventional expert reviews [20]. Previous scientometric investigations on MDS focused on publications up to 2022, establishing useful foundational benchmarks [21, 22]. However, these earlier analyses preceded several transformative advances, including the formal implementation of the IPSS-M molecular scoring system, widespread integration of next-generation sequencing (NGS) into clinical guidelines [6], the emergence of artificial intelligence (AI)-driven prognostic models [23], and clinical adoption of novel agents including venetoclax and luspatercept [12]. These innovations have profoundly reshaped global research priorities and collaborative patterns, highlighting the urgent need for an updated, comprehensive synthesis.

Accordingly, this study provides an updated and comprehensive multi-dimensional scientometric analysis of MDS research published between 2014 and 2025. Specifically, we seek to: (1) map global publication trends and major research contributors; (2) investigate hierarchical collaboration networks at national, institutional, and individual levels; and (3) analyze keyword co-occurrence and reference co-citation clusters to explore core knowledge systems and shifts in research paradigms. This macro-synthesis of the current research landscape is intended to offer evidence-based insights into emerging hotspots and potential research gaps for future MDS studies.

## Methods

### Data origin and retrieval strategy

Data for this study were sourced from the Web of Science Core Collection (WoSCC) [24, 25]. The Science Citation Index Expanded (SCIE), with its rigorous journal selection criteria, was used as the primary subset to ensure high publication quality for subsequent analyses. Retrieval was conducted on August 2, 2025 to collect MDS research from January 1, 2014 to the retrieval date.

Document types were restricted to “article” and “review” as defined by the WoSCC. The language was restricted to English to avoid biases arising from translation discrepancies or classification inconsistencies. To enhance thematic relevance and minimize omissions due to abbreviated titles or overlooked keywords, the retrieval strategy targeted core disease terminology within the abstract, using the following high specificity search query: Abstract=(“Myelodysplastic syndrome” OR “Myelodysplastic syndromes”).

The final curated dataset was used for scientometric analyses. To prevent inconsistencies from variations in keyword spelling, abbreviations, plurals, or synonyms, normalization and synonym merging were performed during the cleaning process. For instance, “MDS” was standardized with “myelodysplastic syndromes”; gene and drug names followed official nomenclature; institutions and countries were harmonized (e.g., merging “Turkiye” and “Turkey” into Turkey). Detailed procedures and their reproducible specific parameters are outlined in **Figure ****S1**.

### Analytical tools and parameters

The scientometric visualization tools VOSviewer (version 1.6.20) [26], CiteSpace (version 6.4.R1) [27] and R-bibliometrix (version 5.2.0) [28] were used in tandem to ensure methodological complementarity. VOSviewer was employed to construct co-authorship, institutional collaboration, and author keyword co-occurrence maps using full counting methods. Node size represented publication volume or frequency of occurrence, while edge thickness reflected link strength. CiteSpace, on the other hand, was primarily utilized for reference co-citation cluster analysis. Parameter settings were optimized to balance analytical breadth and thematic resolution, with cluster labels generated using log-likelihood ratio algorithms for semantic clarity. Node selection was based on the g-index (k = 25), which balanced citation breadth and depth. Modularity (Q) and silhouette (S) values were calculated to assess clustering robustness and reliability. Moreover, given that R-bibliometrix employs different theme detection algorithms as compared to CiteSpace and VOSviewer, R-bibliometrix was applied to perform trend topic analysis on author keywords, thereby enabling a deeper exploration of the structural and temporal dimensions of MDS research themes [28].

## Results

### Annual publication trends

**Fig. 1:**
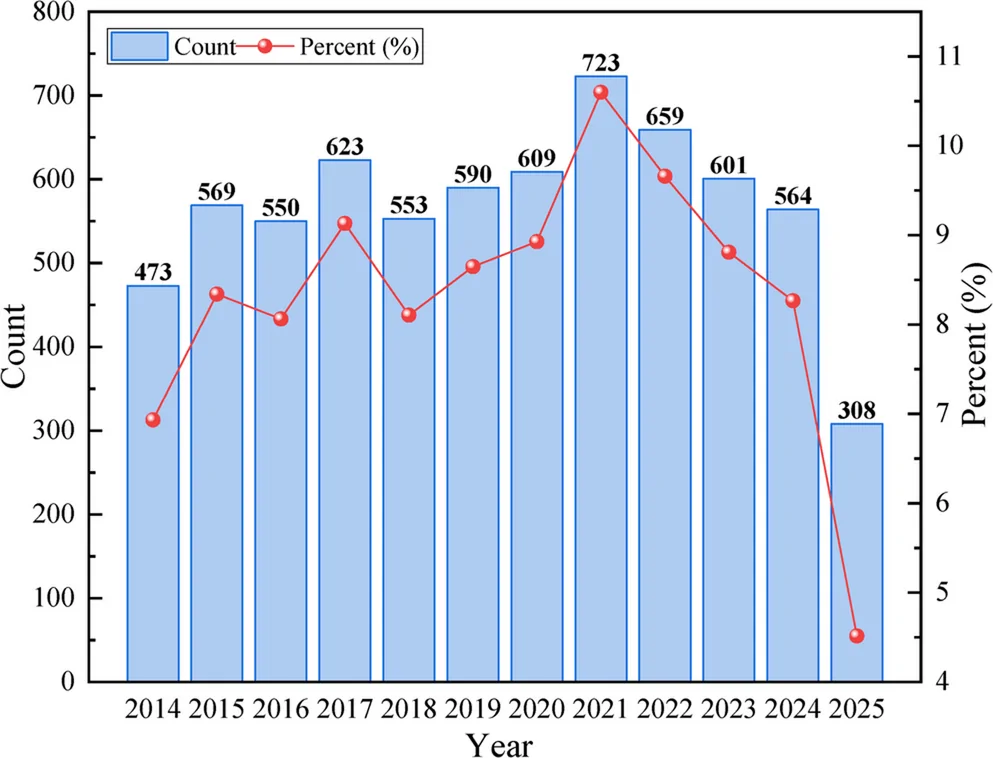
Annual publication numbers for MDS research from 2014 to 2025

Analyzing MDS publications retrieved from WoSCC, the number of yearly publications showed a fluctuating but overall upward trajectory from 2014 (*n* = 473) to a peak in 2021 (*n* = 723) (Fig. 1). Following this peak, annual output entered a phase of decline, decreasing to 659 in 2022, 601 in 2023, 564 in 2024, and further to 308 by August 2025.

### Country/Region contributions and collaboration network analysis

**Fig. 2:**
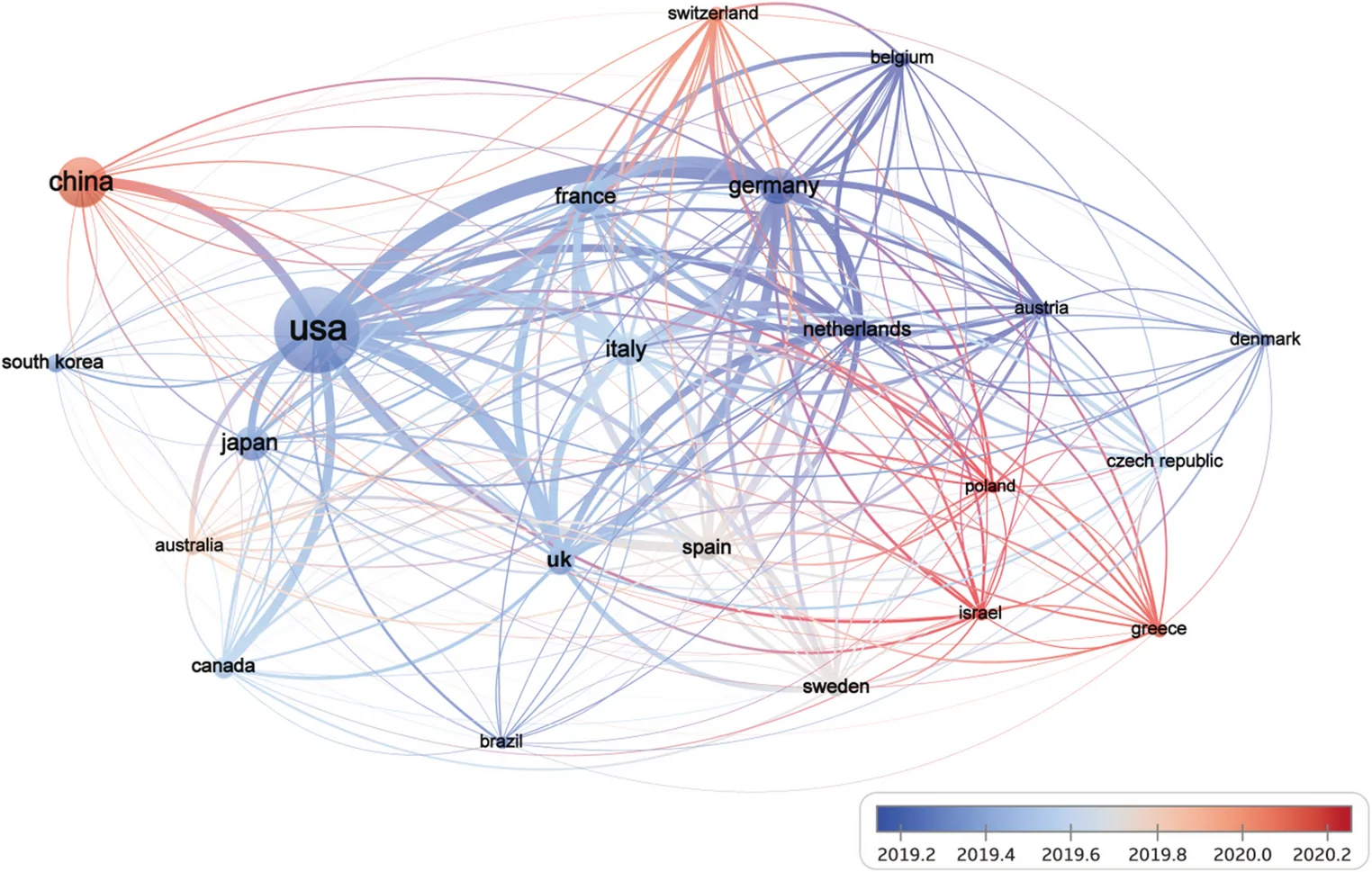
Global collaboration network of prolific countries/regions (threshold = 112, *n* = 22)

A total of 6,822 publications were published from 96 countries and regions. As shown in Table 1, the USA dominated global MDS research with 2,838 publications, followed by China (*n* = 1,169) and Germany (*n* = 717) in second and third place, respectively. The timeline overlay visualization of research activity (Fig. 2) further characterizes collaboration patterns among major nations and reveals different temporal trends in scholarly output. On average, the USA and several key European countries—Germany, Italy, France, and the UK—sustained high and stable research activity in the study period. Larger node sizes and thicker lines confirm their position as the core hubs of global MDS research, with steady publication output and intensive academic collaboration. In contrast, research productivity from several countries like China and Switzerland has increased remarkably in recent years, reflecting their rapid development and growing participation in this research domain.

**Table 1:** The top 10 most productive countries/regions and institutions in MDS research (2014–2025)

Rank	Country/region	Number	Institution	Number
1	USA (North America)	2838	University of Texas MD Anderson Cancer Center (USA)	405
2	China (Asia)	1169	Memorial Sloan Kettering Cancer Center (USA)	215
3	Germany (Europe)	717	Cleveland Clinic (USA)	214
4	Japan (Asia)	661	H. Lee Moffitt Cancer Center and Research Institute (USA)	209
5	Italy (Europe)	601	Dana-Farber Cancer Institute (USA)	204
6	France (Europe)	575	Mayo Clinic (USA)	183
7	United Kingdom (Europe)	453	Harvard Medical School (USA)	149
8	Spain (Europe)	413	Yale University (USA)	135
9	Poland (Europe)	383	Fred Hutchinson Cancer Research Center (USA)	132
10	Netherlands (Europe)	329	University of Washington (USA)	127

### Institution contributions and collaboration network analysis

**Fig. 3:**
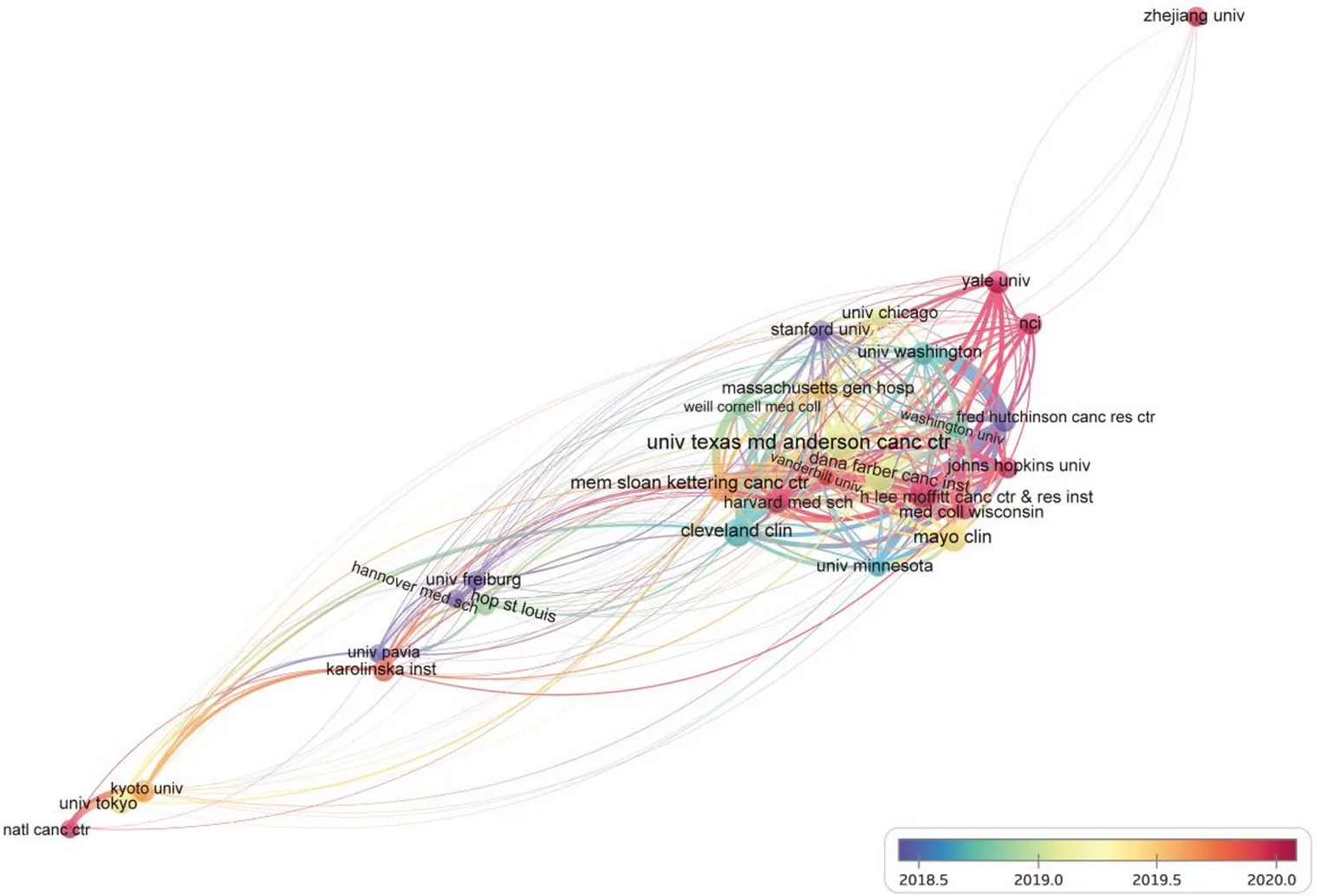
Global collaboration network of prolific institutions (threshold = 73, *n* = 30)

Among the 7,692 identified affiliations, the top 30 institutions each produced more than 73 publications. The institutional landscape of MDS research is highly concentrated in the United States (Table 1). Notably, all top ten institutions by publication output are based in the USA, forming a dominant academic cluster. The University of Texas MD Anderson Cancer Center ranks first with 405 publications, followed by Memorial Sloan Kettering Cancer Center (*n* = 215) and the Cleveland Clinic (*n* = 214). The timeline overlay map of institutional research activity **(**Fig. 3**)** further supports these quantitative findings. Leading USA institutions, including the University of Texas MD Anderson Cancer Center and Memorial Sloan Kettering, have sustained steady and high research output, consolidating their position as stable core contributors to the field. In contrast, some institutions, such as Harvard Medical School (USA), Yale University (USA), and Zhejiang University (China), have witnessed a marked rise in publications in recent years, emerging as fast-growing research hubs. Other institutions, such as the Karolinska Institute (Sweden) and Kyoto University (Japan), exhibit relatively sporadic research activity.

### Core author contributions and co-authorship network analysis

A total of 34,173 authors contributed to MDS-related publications between 2014 and 2025. Inspecting the top 15 most prolific authors reveal a concentrated output landscape where 11 are affiliated with American institutions (Table 2). The University of Texas MD Anderson Cancer Center is the predominant hub, contributing six leading authors, including the top two authors, Garcia-Manero and Kantarjian. Significant contributions also originate from institutions in Germany, France, and Japan.

The co-authorship network (**Figure S2**) resolved into six different clusters, reflecting specialized collaborative groups. The *green cluster*, centered on Garcia-Manero G and Kantarjian HM (University of Texas MD Anderson Cancer Center), drives the core agenda for novel therapeutic strategies in MDS, with a primary focus on hypomethylating agents and advanced combination regimens. The *red cluster* showcases a robust transatlantic network, uniting prominent American and European leaders including Komrokji RS (H. Lee Moffitt Cancer Center and Research Institute), Zeidan AM (Yale Cancer Center), Sekeres MA (University of Miami Miller School of Medicine), Platzbecker U (University Hospital Carl Gustav Carus), and Fenaux P (Saint Louis University Hospital). This cluster has been instrumental in designing and leading pivotal multicenter clinical trials, directly translating research into evolving global treatment protocols. The *purple cluster*, primarily comprising Hasserjian RP and Wang SA (University of Texas MD Anderson Cancer Center), focuses on the morphologic and pathological foundations of MDS. Their work is critical for diagnostic standardization and the continuous refinement of WHO classifications, ensuring the integration of genomic data within a solid clinicopathologic framework. The *yellow cluster* is characterized by a strong regional focus, led by Japanese investigators Atsuta Y, Fukuda T, and Uchida N. Their collaborative work is centered on hematopoietic stem-cell transplantation (HSCT), generating extensive registry-based evidence to optimize clinical applications and outcomes. The *dark blue cluster* represents a cohort of emerging and influential Asian investigators, prominently featuring Chinese scholars Jie Jin (Zhejiang University) and Rong Fu (Tianjin Medical University), whose publications including the bone marrow microenvironment and clinical-translational studies. While demonstrating significant and growing publication output, the international collaborative linkages for this cluster appear less dense compared to the transatlantic core. The *light blue cluster*, surrounding Steensma DP (Harvard Medical School), focuses on the biology and clinical implications of clonal hematopoiesis, representing a niche but influential thematic area within the broader MDS research spectrum.

**Table 2:** Top 15 Most Prolific Authors in MDS Research (2014–2025)

Rank	Author	Institution	Number
1	Garcia-Manero G	University of Texas MD Anderson Cancer Center (USA)	170
2	Kantarjian HM	University of Texas MD Anderson Cancer Center (USA)	117
3	Zeidan AM	Yale Cancer Center, Yale School of Medicine (USA)	108
4	Komrokji RS	H. Lee Moffitt Cancer Center and Research Institute (USA)	102
5	Platzbecker U	University Hospital Carl Gustav Carus (Germany)	95
6	Fenaux P	Saint Louis University Hospital (France)	94
7	Sekeres MA	University of Miami Miller School of Medicine (USA)	85
8	Germing U	Heinrich Heine University Düsseldorf (Germany)	79
9	Steensma DP	Harvard Medical School (USA)	68
10	Ravandi F	University of Texas MD Anderson Cancer Center (USA)	66
11	Jabbour E	University of Texas MD Anderson Cancer Center (USA)	57
12	Padron E	H. Lee Moffitt Cancer Center and Research Institute (USA)	55
13	Atsuta Y	Japanese Data Center for Hematopoietic Cell Transplantation (Japan)	53
14	Patnaik MM	Mayo Clinic (USA)	53
15	Medeiros LJ	University of Texas MD Anderson Cancer Center (USA)	52

### Core journal distribution and impact analysis

The dissemination of MDS research exhibits a well-defined hierarchical structure, primarily concentrated within specialized hematology-oncology journals while demonstrating a broadening reach into interdisciplinary platforms (**Table ****S1**). *Blood* dominates MDS research with 212 publications, solidifying its role as the primary communication hub in the field. *Leukemia*, *Haematologica*, and *The Lancet Haematology* represent the second tier of leading journals, serving as prominent platforms for both mechanistic and clinical investigations. *Blood Advances*, launched in 2016, has quickly established itself as a key open-access platform, reflecting the field’s transition towards more accessible and rapid dissemination of research findings.

Based on citation rankings (**Figure S3**), *Journal of Clinical Oncology*,* Blood*, and *Leukemia* ranked top three in terms of average citation count, underscoring their intellectual prominence and leadership in translational research. Notably, the *Journal of Clinical Oncology* stands out with an impressive average of 132.8 citation count, emphasizing the clinical significance and lasting influence of MDS studies published in major oncology outlets. Beyond the core hematology domain, journals such as *Cancers*, *Frontiers in Oncology*, *Scientific Reports*, and *International Journal of Molecular Sciences* offer interdisciplinary platforms that bridge molecular pathogenesis with bioinformatics, immune microenvironment analyses, and experimental therapeutics. Their inclusion in the co-citation network highlights the expanding interface between basic research and clinical translation.

**Fig. 4:**
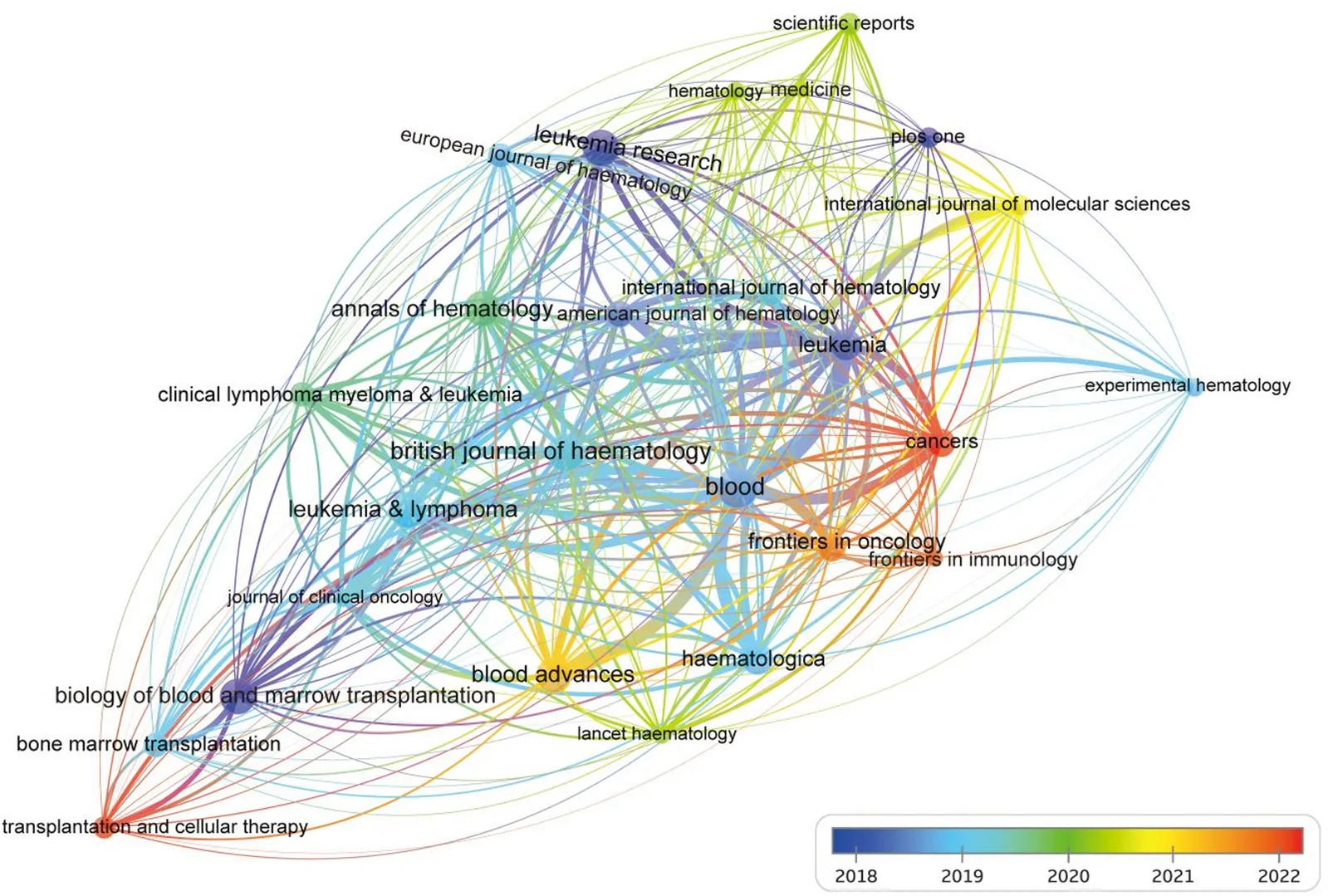
Journal distribution network with timeline overlay (threshold = 54, *n* = 26)

Analysis of the journal distribution network (Fig. 4) identified a dense, interconnected core formed by specialized hematology/oncology journals. This core is surrounded by nodes representing high-impact multidisciplinary journals and journals from adjacent fields such as immunology and transplantation. A timeline overlay map revealed that journals such as *Cancers*,* Frontiers in Oncology*, and *Frontiers in Immunology*, and *Blood Advances* have exhibited heightened publication activity in recent years, as visually reflected in the figure: nodes with warmer color tones correspond to these increasingly active journals. The dual-map overlay (**Figure S4**) further elucidates macro-level knowledge flow. It visualizes three primary citation pathways: citing journals from the “Molecular, Biology, Immunology” and “Medicine, Medical, Clinical” clusters mainly cite publications in the “Molecular, Biology, Genetics” and “Health, Nursing, Medicine” clusters. Among these pathways, two originate from the “Molecular, Biology, Genetics” cluster, this further confirms that current MDS clinical and translational research is deeply rooted in and regularly references foundational literature in molecular biology and genetics.

### Analysis of emerging themes

**Fig. 5:**
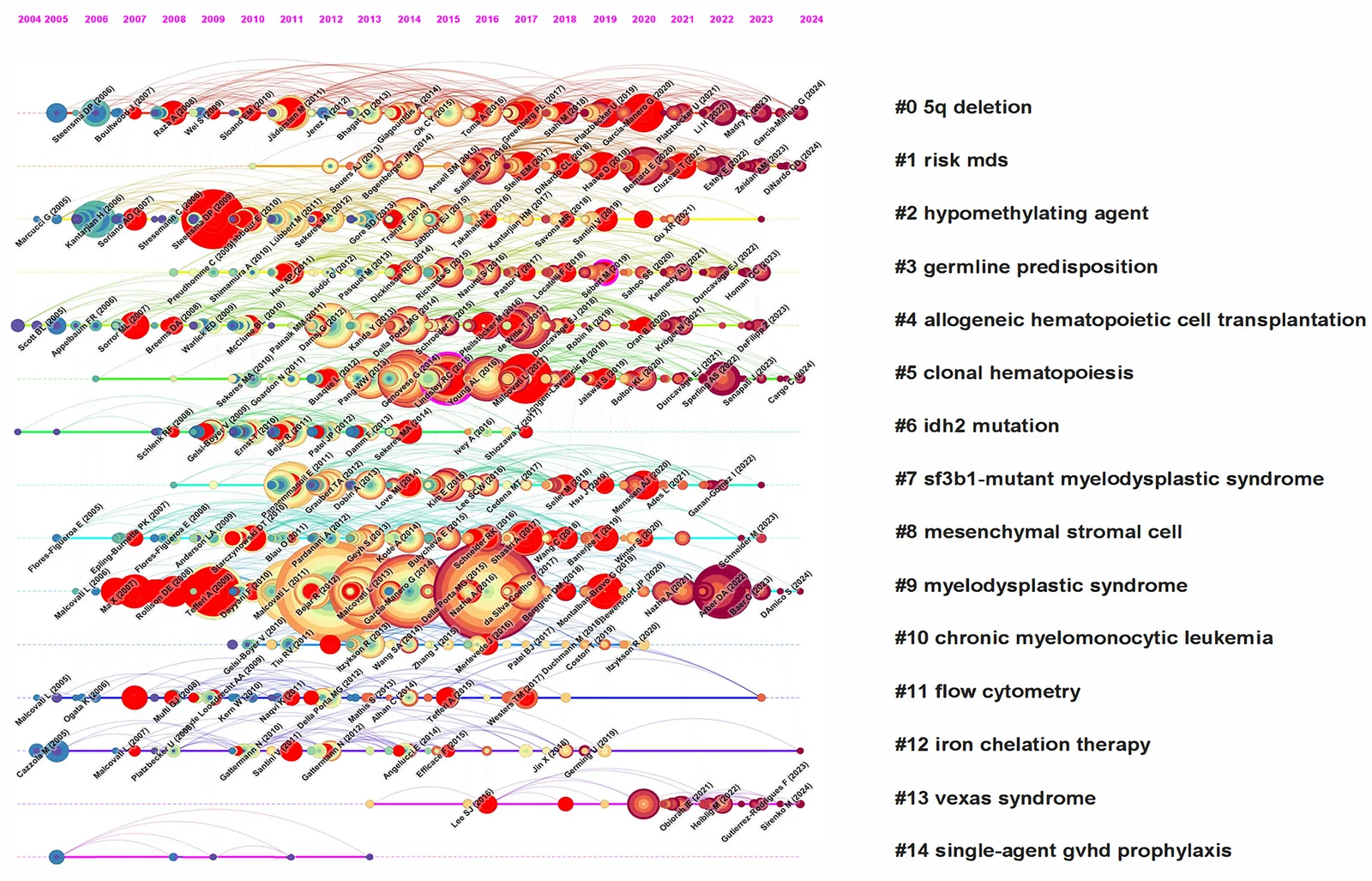
Timeline visualization of the reference co-citation cluster network (link retaining factor = 3.0, look back years = 10, e = 2.0)

To elucidate the intellectual foundation and evolutionary dynamics of MDS research, a reference co-citation cluster analysis was performed. The network generated by CiteSpace exhibited robust structural validity (Q = 0.75; S = 0.91), resulting in the identification of 15 distinct thematic clusters **(**Fig. 5**)**. The core of the knowledge network is formed by long-standing clinical themes, with the three largest clusters being *#0 5q deletion* (121 members), #1 *risk MDS* (120 members), and #2 *hypomethylating agents* (113 members). These clusters collectively constitute the traditional cornerstone of MDS research concerning cytogenetics, prognostic assessment, and first-line therapy. Concurrently, several more recent and reliable (S > 0.85) clusters signal the emergence of cutting-edge directions, such as #3 *germline predisposition*, #5 *clonal hematopoiesis*, and #13 *VEXAS syndrome*.

**Fig. 6:**
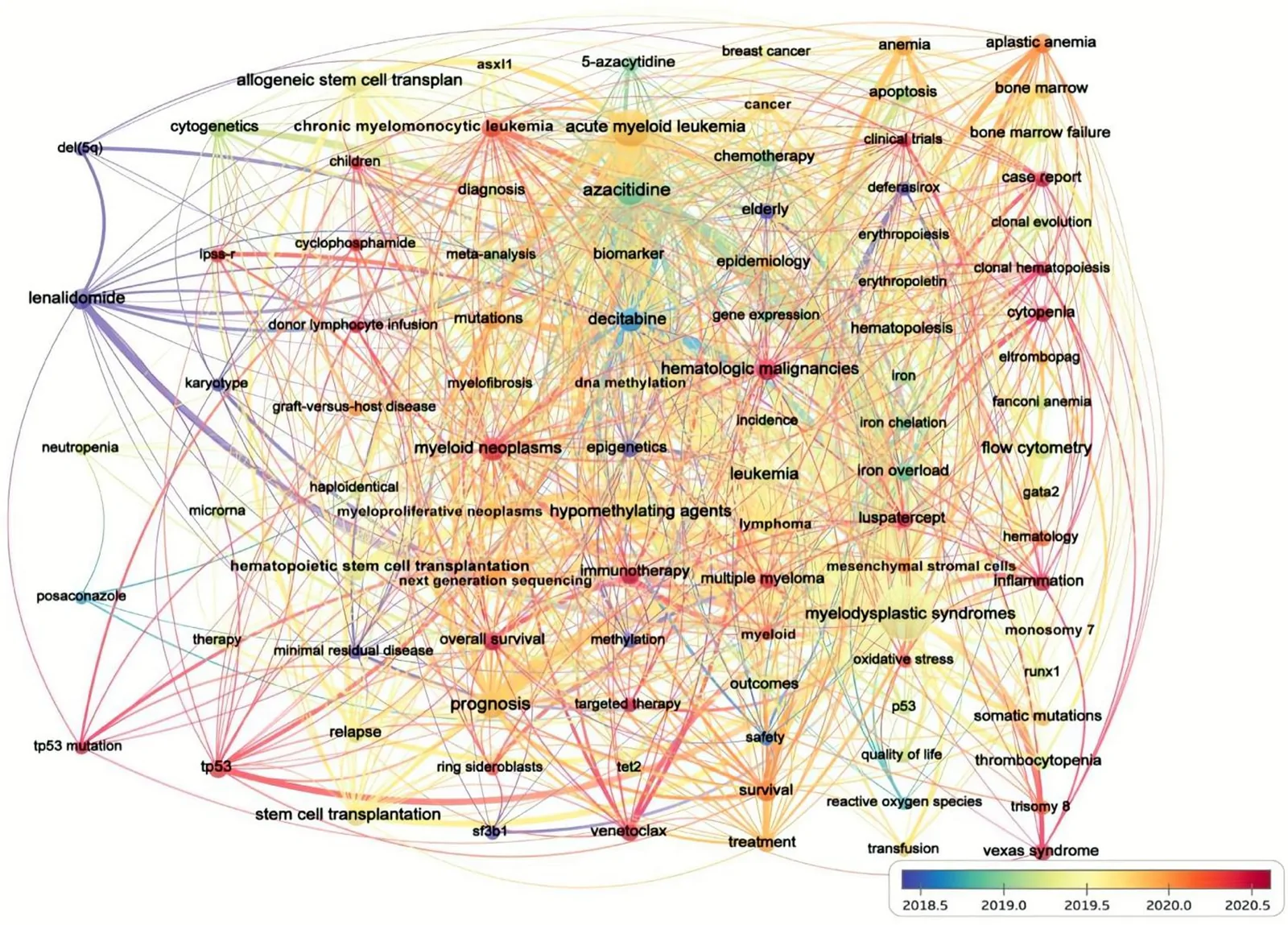
Timeline visualization of standardized keywords co-occurrence network (threshold = 20, *n* = 100)

The timeline overlay visualization of the keyword co-occurrence network **(**Fig. 6**& Table S2)** delineates the temporal dynamics of MDS research across major thematic domains. The color gradient shifting from cool tones to warm tones intuitively illustrates the evolutionary trajectory of research priorities, ranging from foundational classical themes to newly emerging research frontiers.

In the domain of disease progression and prognostic evaluation, the core research hotspots mainly encompass *hematologic malignancies*, *myeloid neoplasms*, *multiple myeloma*, *chronic myelomonocytic leukemia*, *aplastic anemia*, *relapse*, *prognosis*, *survival*, and *overall survival*.

In terms of therapeutic interventions, early research focused predominantly on conventional therapeutic agents including *azacitidine*, *decitabine*, and *hypomethylating agents*, together with *5-azacytidine*, *lenalidomide*, and *chemotherapy*, which are closely associated with early efficacy exploration. Moderate color clusters correspond to supportive care-related themes such as *transfusion* and *erythropoietin*. By contrast, recent research represented by warm color clusters centers on novel therapeutic strategies, prominently including *venetoclax*, *targeted therapy*,* luspatercept*, and *immunotherapy*.

The cluster of transplantation and hematologic manifestations covers key hotspots such as *allogeneic stem cell transplantation*, *hematopoietic stem cell transplantation*, *graft-versus-host disease*, *donor lymphocyte infusion*, *anemia*, *neutropenia*, and *thrombocytopenia*.

The molecular and pathological mechanism cluster comprises core keywords including *DNA methylation*, *TP53*, *TP53 mutation*, *TET2*, *ASXL1*, *RUNX1*, *GATA2*, *clonal evolution*, *epigenetics*, *SF3B1*, *somatic mutations*, *clonal hematopoiesis*, *del(5q)*, *monosomy 7*, *trisomy 8*, *oxidative stress*, *inflammation*, and *ring sideroblasts*. Furthermore, *NGS*, *flow cytometry*, and *cytogenetics* serve as pivotal technical tools for etiological and phenotypic research of MDS.

From the perspectives of clinical research and epidemiology, representative hot keywords include *meta-analysis*, *case report*, *clinical trials*, *epidemiology*, *incidence*, *children*, *quality of life*, *IPSS-R*, *safety*, and *cyclophosphamide*, as well as MDS-related comorbidities typified by *VEXAS syndrome.*

To further validate and supplement the analytical results of the above research themes, we performed a topic trend analysis based on author’s keywords using the R-bibliometrix (**Figure S5 & Table S3**). The outcomes were broadly consistent with those generated by VOSviewer and CiteSpace. Consistently prominent trending topics included *del(5q)*, *venetoclax*, *azacytidine*, *decitabine*, *prognosis*, *inflammation*, *VEXAS syndrome*, and *overall survival*, etc. In addition, the analysis identified an obvious emerging research direction: the increasing application of *AI* and *machine learning* in MDS research.

## Discussion

This study analyzed 6,822 publications on MDS published between 2014 and 2025, aiming to provide a comprehensive overview of the global evolutionary trajectory, structural characteristics, and thematic shifts in this research field. Time-trend analyses demonstrated that following a prolonged period of rapid growth, MDS research has entered a phase of relative consolidation since approximately 2021. The expansion of MDS research activity may be mainly attributed to the development and widespread clinical application of critical tools (e.g., NGS, IPSS-R, IPSS-M) and therapeutic agents (e.g., venetoclax). Specifically, NGS, which substantially expanded the understanding of MDS genomic landscapes and directly facilitated major updates in diagnostic and prognostic frameworks [29, 30]. Notably, the IPSS-M incorporated somatic mutation data to significantly improve risk stratification: it enabled reclassification of approximately 42%−46% of patients and demonstrated superior performance compared to the IPSS-R in predicting leukemia-free survival and overall survival [6, 31]. Concurrently, the ICC redefined the blast threshold for AML from ≥ 20% to ≥ 10% in genetically defined cases, delimiting the diagnostic boundary between MDS and AML—an advancement that aligns with the thematic shifts identified in this study [4]. As these foundational frameworks became established, research attention gradually shifted toward more refined, biologically driven, and clinically integrated questions, as reflected by the emergence of key topics such as variant allele frequency (VAF), clonal dynamics, venetoclax-based regimens, and machine learning-related analytical approaches [13, 32].

Global collaboration network analysis revealed a pronounced core-periphery structure, with the United States and Western Europe positioned at the center. These regions contributed the largest volume of publications and maintained the most extensive international collaborative networks, which may be associated with their solid research strength, cutting-edge technologies and abundant research funding [33, 34], thereby shaping global research priorities and clinical standards. Despite the rapidly increasing publication output from Asian countries—particularly China—their involvement in high-impact global collaborative networks remains limited. This imbalance indicates that the current evidence base for MDS research is still predominantly derived from Western populations. Previous studies have highlighted that geographic and ethnic disparities in genomic data representation may compromise the performance of molecular prognostic models in diverse clinical settings [35–37]. For instance, Jiang et al. reported significant differences in cytogenetic and molecular genetic features between Asian and Western patients with MDS: del(5q), TET2, and SF3B1 mutations were more common in Western cohorts, whereas trisomy 8, del(20q), and U2AF1 mutations were more frequent in Asian cohorts [38]. Such population-specific mutational patterns may influence the calibration and applicability of IPSS-M in non-Western populations. Indeed, when Gill et al. externally validated IPSS-M in an independent Asian cohort, its C-index for overall survival was only 0.57, whereas their newly developed Asian Prognostic Scoring System (APSS) achieved a C-index of 0.73, demonstrating substantially better performance in this population [23].

Intellectual theme analyses suggest that MDS research is gradually advancing toward precision medicine, though the full integration of this paradigm into routine clinical practice still requires time. To date, the molecular understanding of the genetic and epigenetic architecture of MDS has become increasingly refined [32]. Early studies mainly focused on TP53 mutations as biomarkers of high-risk MDS, while subsequent investigations expanded to cover epigenetic regulators such as TET2 and ASXL1, transcription factors including RUNX1 and GATA2, and splicing-related genes represented by SF3B1^29^. This evolving molecular cognition reflects a critical conceptual shift: research has moved beyond single-driver mutation models and begun to regard clonal evolution and molecular heterogeneity as core determinants of disease progression and therapeutic resistance. Beyond genetic aberrations, inflammation has been redefined from a mere epiphenomenon to a central pathogenic driver of MDS, standing out as a prominent research hotspot validated by the keyword analysis [39]. As the pathogenic microenvironment for MDS initiation and development, chronic inflammation facilitates clonal origination and disease progression, and forms a vicious cycle with genetic abnormalities; meanwhile, inflammatory status also serves as a vital prognostic indicator, making targeted anti-inflammatory therapy a promising therapeutic direction [40]. Notably, the prevalence of MDS in patients with VEXAS syndrome has been estimated to range from 25% to 55% [41], a late-onset autoinflammatory disorder driven by somatic mutations, which perfectly illustrates the interplay between inflammation and MDS, whereby somatic UBA1 mutations directly link systemic inflammation to myelodysplasia [42]. Additionally, the obvious distinction between “children” and “elderly” groups in the keyword co-occurrence network **(**Fig. 6**)** may imply inherent age-related biological disparities, suggesting that pediatric MDS has become a rising research focus and highlighting the necessity of adopting age-stratified strategies in both MDS research and clinical management [43].

Research on HMAs and conventional prognostic scoring systems may still dominate the overall MDS research landscape, which largely reflects the inherent limitations of existing therapies rather than insufficient scientific innovation [12]. Clinically, azacitidine can induce therapeutic responses in high-risk MDS patients, but the responses are poorly durable with a median duration of around 10 months, and most patients eventually suffer from relapse or progression to AML [11]. Such clinical dilemmas are particularly prominent in high-risk molecular subtypes, especially TP53-mutated MDS characterized by severe genomic instability and inherent treatment resistance [44]. The rapid development of NGS, the exploration of somatic mutations and clonal hematopoiesis, and the recognition of key cytogenetic abnormalities (including del(5q), monosomy 7, trisomy 8 and ring sideroblasts) have jointly established an integrative genomic-cytogenetic paradigm, which underpins modern MDS classification systems and molecular prognostic tools such as IPSS-M, while also revealing their limitations in population generalizability and dynamic risk evaluation [6]. Even so, traditional treatment regimens and classic risk stratification systems still occupy a fundamental position in clinical decision-making [12]. Constrained by the bottlenecks of conventional therapies, increasing attention has been paid to novel targeted and individualized strategies: luspatercept can effectively improve transfusion independence in low-risk transfusion-dependent MDS patients [45], while venetoclax-based combination regimens have been widely investigated for relapsed and refractory high-risk MDS [46, 47].

Despite the emergence of novel therapies, clinical outcomes in MDS are constrained by inherent temporal heterogeneity. As a clonally driven and dynamically evolving disease, MDS is accompanied by continuous clonal evolution and fluctuating VAF. Nevertheless, these dynamic biological characteristics have not yet been systematically incorporated into standard clinical decision-making, thereby limiting their translational application [14]. Although this study cannot directly elaborate on the specific molecular mechanisms, theme analysis identified some keywords related to relapse and AML with mainstream therapeutic terms. This provides suggestive evidence on secondary drug resistance, which represents a critical bottleneck in MDS clinical management given that therapeutic resistance frequently induces disease relapse, and inadequate disease control further promotes progression to AML. This finding is consistent with clinical observations that even HMA-venetoclax combinations, despite high initial response rates, cannot avert eventual disease progression in a substantial proportion of patients with high-risk MDS [48]. Furthermore, immunotherapy has emerged as a promising research frontier, but its large-scale clinical application still faces considerable challenges, including CAR-T therapy [49].

Lastly, AI and machine learning have flourished across various medical fields, and MDS are no exception, as supported by the findings of trend topic analysis. Multimodal data integration (encompassing genomic, cytogenetic, morphological, clinical, and longitudinal follow-up data) shows great potential in MDS for auxiliary diagnosis, intelligent risk stratification, prognosis prediction, therapeutic response forecasting, drug resistance identification, and dynamic monitoring of clonal evolution, ultimately facilitating individualized and precise clinical management [50, 51].

This scientometric study has several prominent strengths. First, systematic literature screening and standardized synonym processing were adopted to minimize retrieval bias and improve data consistency, which lays a solid foundation for exploring the knowledge structure of MDS research. Academically, this method provides a reproducible analytical framework to identify research gaps, track emerging research hotspots, and guide future research priorities. Clinically, the integrated overview of the research landscape can clarify the orientation of translational medical resource allocation and facilitate the development of equitable precision medicine.

Nonetheless, several limitations should be noted, consistent with prior scientometric studies [52, 53]. First, the search strategy was primarily developed using the MeSH term “myelodysplastic syndromes” from PubMed and did not incorporate the revised WHO terminology “myelodysplastic neoplasm(s)” [47], given that this revised term has not yet gained widespread acceptance [48]. Future updates to this study may be warranted as the revised terminology becomes more broadly adopted. Second, reliance on the WoSCC may have resulted in the omission of relevant studies indexed in other databases. Third, scientometric approaches are limited to macro-level trend analysis and cannot directly capture biological mechanisms or clinical nuances.

## Conclusion

MDS research is witnessing a gradual shift towards precision medicine. Future progress will likely depend on three key directions. First, building collaborative platforms with diverse multi-ethnic cohorts to validate and optimize molecular classification and prognostic tools, which can alleviate the imbalance of population representation and improve the generalizability of precision medicine. Second, systematically exploring the mechanisms of genomic instability and therapeutic resistance to identify novel targets for refractory disease, providing theoretical evidence for overcoming relapse, progression and drug resistance. Third, exploring rational combination and sequential regimens integrating novel agents, such as venetoclax, with standard therapies and personalized supportive care to improve patients’ long-term prognosis and quality of life. Strengthening cross-regional and multidisciplinary cooperation among clinicians, pathologists, basic/clinical researchers, and AI scientists is also essential to facilitate clinical translation. Collectively, these efforts will drive global MDS diagnosis and treatment toward higher precision, equity and clinical practicality.

## Supplementary Information

Below is the link to the electronic supplementary material.

Supplementary Material 1

## Data Availability

The datasets generated and analyzed during this study are available from the corresponding author upon reasonable request. The original data were derived from the Web of Science Core Collection (https://www.webofscience.com), which is a subscription-based database. The search was conducted on August 2, 2025, using the query detailed in the Methods section, ensuring the reproducibility of the dataset.
