# Supplementary material for: Mapping the research landscape of myelodysplastic syndromes: a scientometric analysis (2014–2025)
Source: Clin Exp Med. 2026 Jun 25;26(1):326. doi: 10.1007/s10238-026-02226-z (PMC13562280; doi:10.1007/s10238-026-02226-z)
Supplement: Supplementary file 1 [file 10238_2026_2226_MOESM1_ESM.pdf]

Supplementary Tables

Table S1. Top 15 core journals of MDS research ranked by number of publications

Rank	Journal	JCR_{2024}	IF_{2024}	Number
1	Blood	Q1	23.1	212
2	British Journal of Haematology	Q1	3.8	188
3	Leukemia & Lymphoma	Q3	2.2	177
4	Leukemia Research	Q3	2.2	174
5	Annals of Hematology	Q2	2.4	169
6	Blood Advances	Q1	7.1	168
7	Biology of Blood and Marrow Transplantation*	Q2	4.3	165
8	Leukemia	Q1	13.4	144
9	Haematologica	Q1	7.9	142
10	Frontiers in Oncology	Q2	3.3	118
11	Cancers	Q2	4.4	116
12	American Journal of Hematology	Q1	9.9	112
13	International Journal of Hematology	Q1	1.8	103
14	European Journal of Haematology	Q2	2.3	96
15	Clinical Lymphoma, Myeloma & Leukemia	Q2	2.7	92

*Impact factor (IF) and quartile data are sourced from Journal Citation Reports (JCR) 2022.

Table S2. Top 30 keywords ranked by occurrence frequency via VOSviewer

Keywords	Occurrence frequency
myelodysplastic syndromes	2872
acute myeloid leukemia	896
hematopoietic stem cell transplantation	420
azacitidine	311
prognosis	253
myeloid neoplasms	245
hypomethylating agents	184
leukemia	166
decitabine	154
survival	138
flow cytometry	102
hematological malignancies	100
lenalidomide	95
mutation	88
chronic myelomonocytic leukemia	84
anemia	80
iron overload	80
cancer	79
treatment	79
aplastic anemia	76
cytogenetics	67
bone marrow	66

relapse	66
apoptosis	64
tp53	62
chemotherapy	58
dna methylation	58
diagnosis	56
bone marrow failure	54
immunotherapy	51
thrombocytopenia	50
inflammation	49
venetoclax	49

Table S3. Trend topics analysis via R-bibliometrix

Term	Frequency	Year (Q1)	Year (Median)	Year (Q3)
prognostication	16	2015	2016	2020
rituximab	13	2015	2016	2020
epigenetics	45	2015	2017	2022
del(5q)	24	2015	2017	2020
lenalidomide	95	2016	2018	2021
methylation	91	2016	2018	2022
azacytidine	311	2016	2019	2022
decitabine	154	2016	2019	2021
hematopoietic cell transplantation	432	2017	2020	2022
prognosis	253	2017	2020	2023
overall survival	138	2018	2021	2023
next generation sequencing	105	2018	2021	2022
inflammation	49	2020	2022	2024
case report	39	2021	2022	2023
venetoclax	49	2021	2023	2024
vexas syndrome	47	2022	2023	2024
machine learning	13	2021	2024	2024
artificial intelligence	12	2022	2024	2024

Supplementary Figures

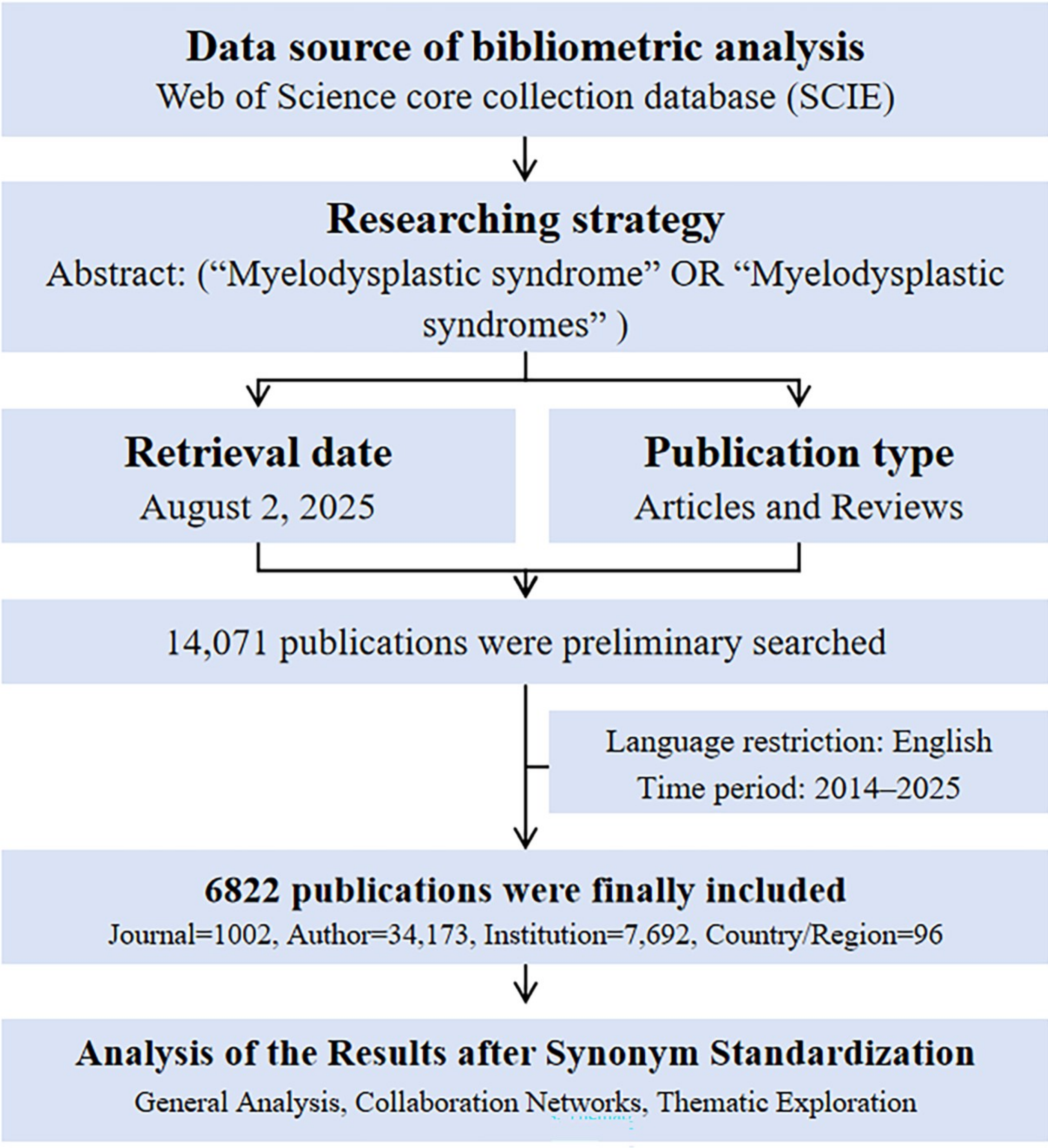

Figure S1. Study flowchart.

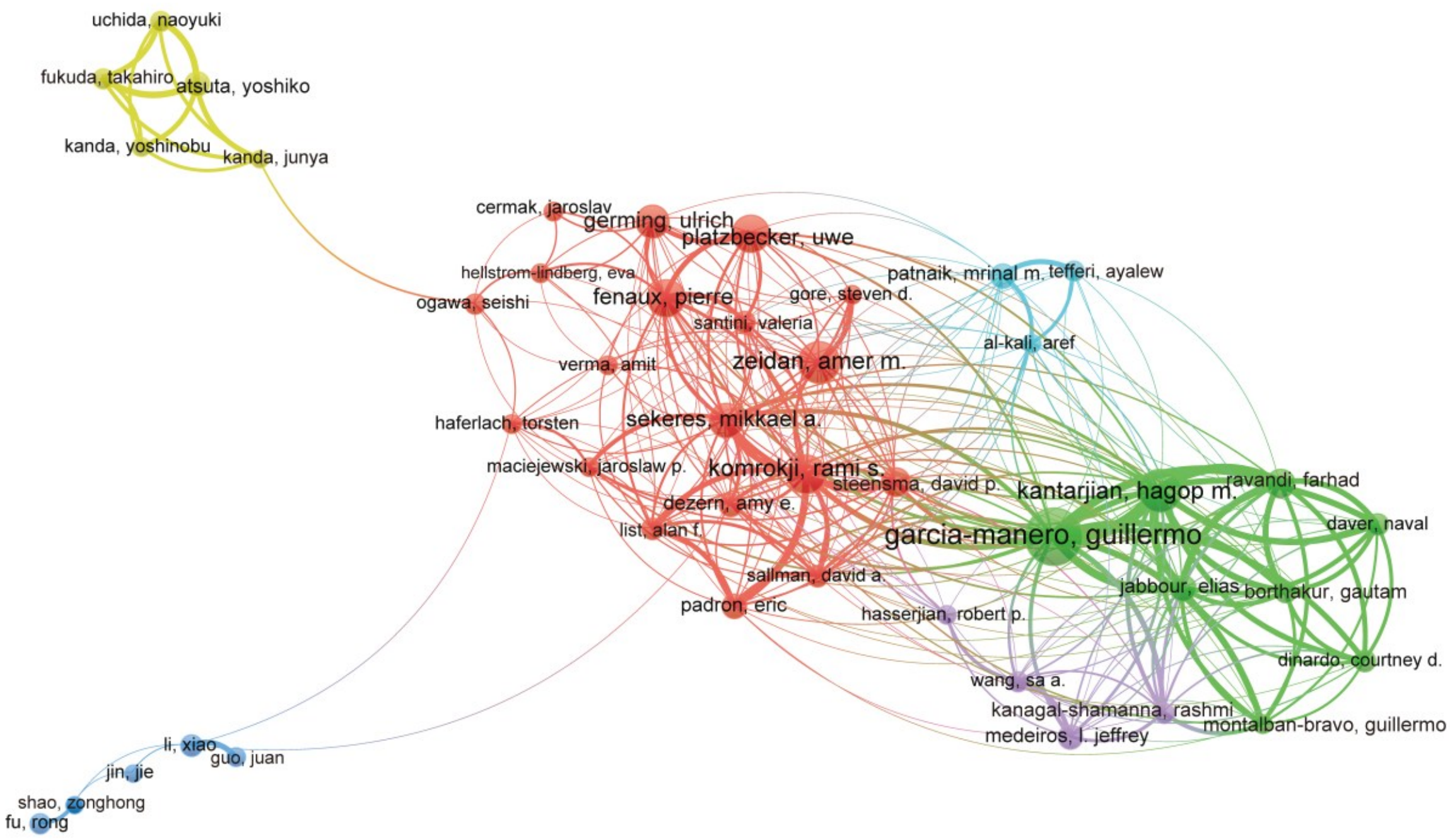

Figure S2. Global collaboration network of prolific authors (threshold=34, n=44)

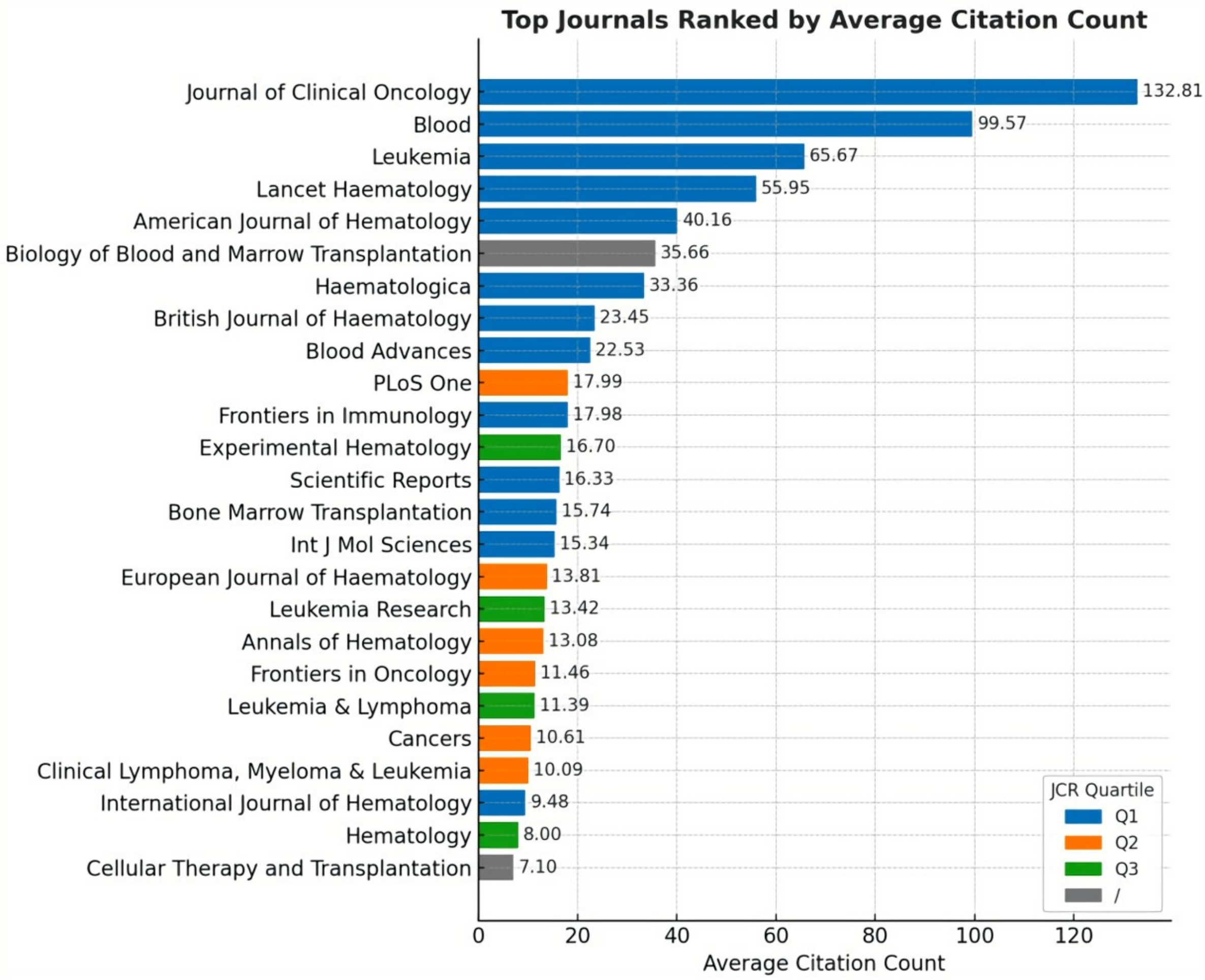

Figure S3. Top journals ranked by average citation count

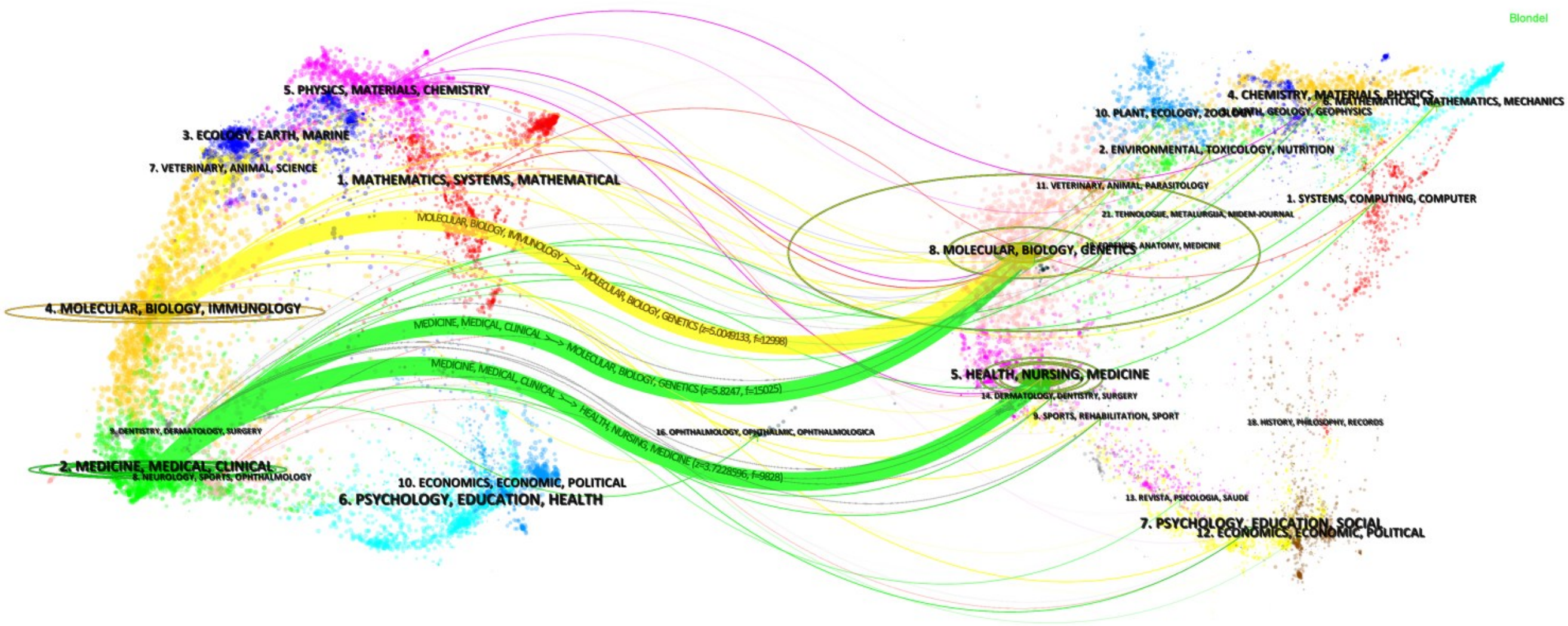

Figure S4. Dual-map overlay of journal citations featuring clusters of citing journals on the left and clusters of cited journals on the right

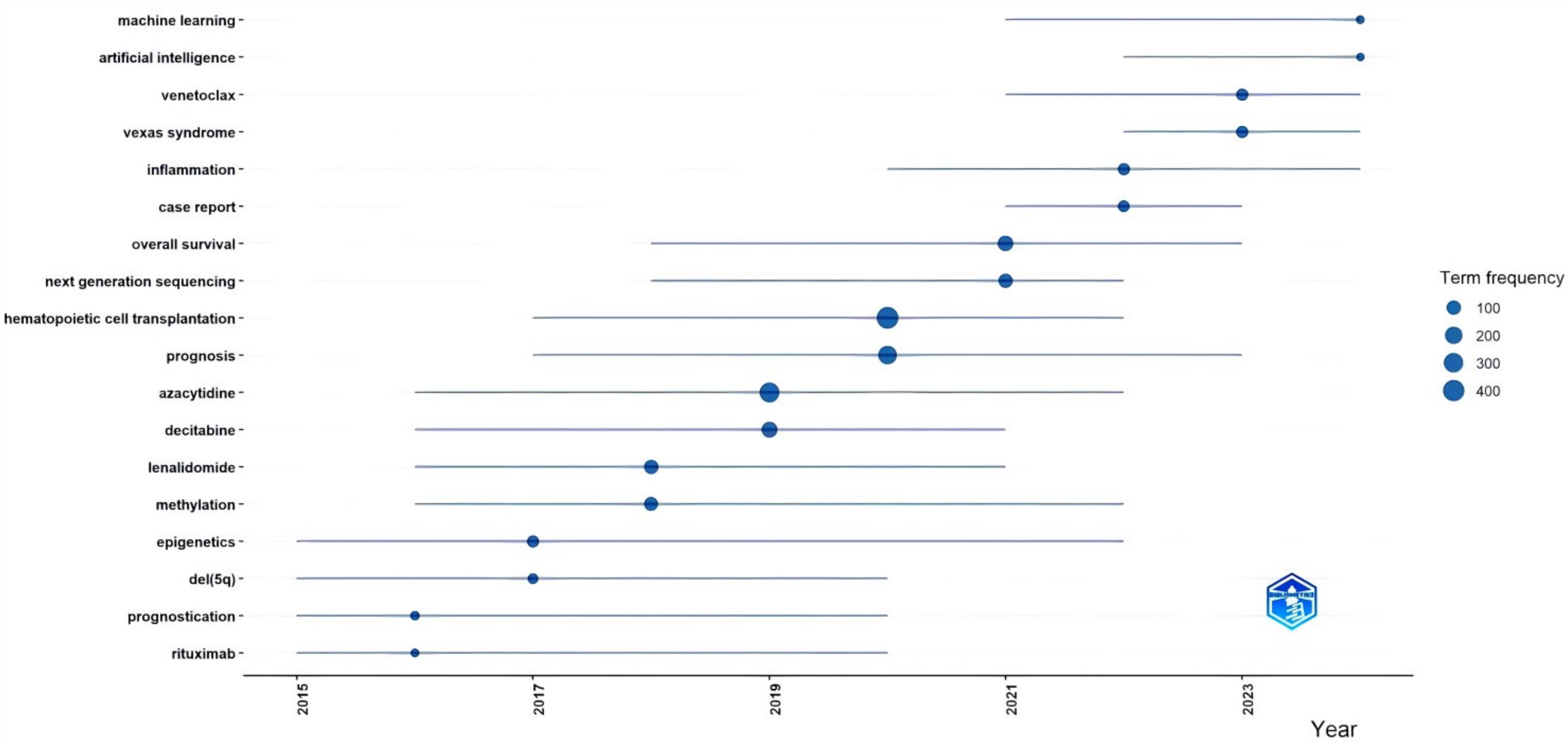

Figure S5. Trend topics of standardized keywords (timespan=2014 to 2025, word minimum frequency=12, number of words per year=2).
